# The emergence of Clade IIb and Ib mpox viruses: a state-of-the-art review

**DOI:** 10.1097/MS9.0000000000004534

**Published:** 2025-12-11

**Authors:** Toufik Abdul-Rahman, Jann Ludwig Mueller-Gomez, Hala Ibrahim Thaalibi, Innocent Ayesiga, Oyinbolaji Akinwande Ajetunmobi, Aderinto Nicholas, Godfred Yawson Scott, Lukman Lawal, Muritala Adewale Lawal, Selimat Ibrahim

**Affiliations:** a Department of Research, Toufik’s World Organization, Sumy, Ukraine; b Center for Research in Health Sciences (CICSA), Faculty of Medicine, Anahuac University North Campus, Huixquilucan, Mexico; c Faculty of Medicine, Beirut Arab University, Beirut, Lebanon; d Research Department, Ubora Foundation Africa-Uganda, Kampala, Uganda; e Department of Public and International Affairs, University of Lagos, Akoka, Nigeria; f Department of Medicine and Surgery, Ladoke Akintola University of Technology, Ogbomoso, Nigeria; g Department of Medical Diagnostics, Kwame Nkrumah University of Science and Technology, Ghana; h Nuffield Department of Population Health, University of Oxford, Oxford, UK; i Department of Emergency Medicine, Lagos State University Teaching Hospital, Lagos, Nigeria; j Department of Clinical Trials, Center for Malaria and Other Tropical Diseases, Ilorin, Kwara State, Nigeria

**Keywords:** Clade Ib, Clade IIb, monkeypox, mpox, orthopoxvirus

## Abstract

Mpox, a zoonotic Orthopoxvirus infection, was historically endemic to West and Central Africa. In 2022, it sparked a significant global outbreak, raising concerns about its spread beyond traditional regions. Notably, the emergence of Clade IIb and Ib strains has alarmed the global health community due to their increased transmission among humans. This paper examines mpox viruses and their general characteristics, their evolution, and the emergence of novel clades with unique features. Clade IIb stands out for its unique genetic mutations, which may enhance its transmissibility. Clinically, infections often present with fewer anogenital lesions and milder symptoms, which can complicate diagnosis. PCR testing remains the most reliable diagnostic tool, although emerging point-of-care tests show promise. Treatments such as tecovirimat and immune-based therapies (e.g., VIGIV) have demonstrated success, despite limitations. The MVA vaccine provides partial protection, although its efficacy against Clade IIb requires further validation. The emergence of Clades IIb and Ib, with distinct transmission dynamics, especially among specific population groups, underscores the need for targeted public health responses. This review highlights critical surveillance challenges in endemic African regions, where underreporting and limited diagnostic infrastructure hinder early detection. By analyzing the evolution and spread of newer clades, this review provides unique insights into mpox’s shifting epidemiology and outlines implications for global preparedness, including the urgent need for strengthened surveillance systems, equitable access to vaccines, and coordinated international efforts focusing on research and preparedness to help mitigate future risks.

## Introduction

Currently, the world is facing a resurgence of the mpox virus with a significant impact on healthcare systems and individuals. The virus and the disease it caused were named upon discovery in 1958, before the current best practices in naming viruses and diseases were established. Historically, the Congo Basin and West African mpox clade variants were identified based on their spatial distribution[1]. However, to ensure that adopted names for the virus, its variants, and the disease it causes align with current best practice, the World Health Organization (WHO) organized an *ad hoc* expert meeting on 8 August 2022 to discuss (mpox virus) MPXV clade characteristics and suggest names for them. The meeting discussed a neutral naming scheme while reviewing the phylogeny and characteristics of MPXVs. The Congo Basin clade was designated Clade I following a consensus. In contrast, the West African clade was designated Clade II, comprising the two phylogenetically divergent subclades, IIa and IIb (initially known as Clade III)[1]. A year after the meeting, a divergent lineage of Clade I was discovered, which was named Clade Ib; the former Clade I was then renamed Clade Ia[2].

**HIGHLIGHTS**

- The world is currently experiencing an mpox outbreak with a significant impact on healthcare systems and individuals.
- The emergence of Clades II and Ib has created a spatial–temporal shift of the mpox virus.
- Continuous genomic monitoring is crucial to identify any genetic changes that may impact public health responses.

Although Clade IIb has been identified as the primary cause of the current global outbreak, which began in 2022, infections with Clades Ia and Ib are being recorded in endemic areas, with Clade Ib showing a strong potential to spread rapidly across borders, similar to IIb. By 12 July 2023, the Clade IIb MPXV had affected 112 countries across all WHO regions, with 88 288 laboratory-confirmed cases and 149 deaths reported[3]. Among cases with available information, 96% are males with a median age of 34 years. Only 1% of cases were children or adolescents. Of those who reported sexual orientation, 87% self-identified as gay, bisexual, or other men who have sex with men. Additionally, 48% of cases with known HIV status were HIV-positive. The most common mode of transmission was sexual contact (69%). The case-fatality ratio was 0.17%, which is significantly lower than previous estimates for other mpox clades[3].

Clade Ib is also virulent and has been predominant in the Northern and Southern Kivu Provinces of the Democratic Republic of Congo (DRC), with sustained human transmission[4]. Over 108 Clade Ib cases had been confirmed in DRC between September 2023 and January 2024. About 48.1% of these cases were male, and 29% were sex workers, with the overall median age of the patients at 22 years[5]. One of the predominant spreads of this strain is through sexual intercourse, since it has been identified on penile and vaginal swabs. By August 2024, multiple cases of Clade Ib had been identified in Uganda, Burundi, Rwanda, and Kenya. Two Clade Ib cases were reported in Uganda, while four cases had been identified in Rwanda[6]. By January 2025, Burundi had reported over 299 cases of Clade Ib, arising from human-to-human transmission, with children under 5 years old accounting for 38% of the total cases[4]. These cases had a variable spatial–temporal distribution, with the majority of cases being reported from the western part of Bujumbura Mairie Province[7].

The emergence of Clade IIb and Ib mpox highlights the significance of surveillance for infectious diseases and their potential for rapid cross-border dissemination. This manuscript provides a comprehensive overview of the Mpox viruses and their general characteristics, their evolution, and the emergence of novel clades with unique features, as well as future directions, along with the role of genomic surveillance in overcoming the current outbreak. This study complies with the Transparency in the Reporting of Artificial Intelligence (TITAN) Guidelines 2025[8].

## Methodology

Studies included in this review were sourced from multiple academic databases, including PubMed, Scopus, Embase, and the Cochrane Library. A comprehensive search strategy was developed using a range of relevant keywords, including “mpox,” “monkeypox,” “orthopoxvirus,” “poxvirus infection,” “Clade IIb,” “Clade Ib,” “virus evolution,” “genetic mutations,” “phylogenetics,” “transmission,” “clinical features,” “anogenital lesions,” “mild symptoms,” “diagnosis,” “point-of-care testing,” “tecovirimat,” “VGIV,” “antiviral agents,” “MVA vaccine,” “vaccination,” “outbreak,” “surveillance,” “public health,” “zoonotic disease,” and “emerging infectious diseases.” Boolean operators such as AND and OR were used to combine and refine these terms during the search process. The review considered various study designs, including cross-sectional studies, experimental studies, systematic reviews, and scoping reviews. Additional data were obtained from international health organizations such as the WHO and the Africa Centres for Disease Control and Prevention (Africa CDC) to ensure that the latest developments on the topic were captured. Only studies published in English were included; studies published in other languages were excluded.

## Taxonomic classification and related viruses

**Figure 1.**
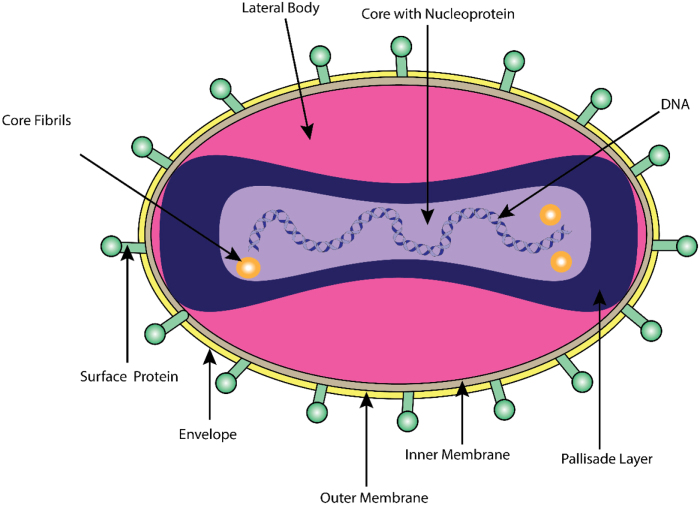
Structure of the mpox virus. The figure illustrates the microstructure of the mpox virus with emphasis on the different proteins, especially those involved in its pathogenesis.

The MPXV is a large double-stranded DNA virus belonging to the Orthopoxvirus genus, characterized by a genome size of approximately 197 kilobases (kb), encoding roughly 190 open reading frames^[9,10]^. Structurally, MPXV virions are brick- or ovoid-shaped, measuring 200–250 nm, and enclosed within a geometrically corrugated lipoprotein outer membrane, akin to other Orthopoxviruses (Fig. 1). Each virion is composed of several distinct structural components, including an outer membrane, surface tubules, two lateral bodies, and the linear DNA genome[9].

MPXV is also part of the Poxviridae family, subfamily Chordopoxvirinae^[11–13]^. The Orthopoxvirus genus contains several viruses that affect humans and multiple wild or domestic mammals, including Variola virus (smallpox), Vaccinia virus (used in smallpox vaccines), Cowpox virus, Camelpox virus, Horsepox virus, and Borealpox virus^[11–14]^. Among these, cowpox virus has the most conserved genome, while other Orthopoxviruses contain point mutations, deletions, and rearrangements that differentiate them from the original progenitor CPXV sequence[13]. MPXV is more closely related to Vaccinia virus and certain Cowpox virus strains than to Variola virus[15]. The classification of poxviruses into genera is based on shared antigenic similarity, induction of immunological cross-protection, and phylogenetic grouping[12].

## Historical perspective: name changes and outbreak timeline

### Origin and first case of mpox

Mpox was first discovered in 1958 during vaccine research involving cynomolgus monkeys at a research institute in Copenhagen, Denmark^[11,16]^. Initially named “monkeypox,” the name was a misnomer since it was based on the host species rather than the true reservoir of the virus. The actual source of the disease remains unknown, although African rodents and nonhuman primates are potential reservoirs that can transmit the virus to humans[17].

The first human case of mpox was identified on 1 September 1970, when a 9-month-old child was admitted to a Hospital in the DRC, then known as the Republic of the Congo^[11,12,14]^. Initially suspected to have smallpox, laboratory tests revealed that the infection was not caused by the Variola virus but by a virus previously isolated during a pustular disease outbreak in captive macaques in Denmark. This discovery led to the naming of the infection as “monkeypox”^[18,19]^.

Between October 1970 and May 1971, six human cases of mpox were reported in Liberia, Nigeria, and Sierra Leone[12]. From the 1970s to the late 1980s, sporadic cases were documented among both humans and animals in the forested regions of West and Central Africa, establishing these areas as endemic regions with fluctuating numbers of reported cases over the years^[20–22]^. Specifically, between 1980 and 1985, 282 cases were reported in Zaire, now known as the DRC[11].

In 2003, the first case of mpox outside Africa was documented in the USA. This was recorded following the importation of rodents from Ghana as pets, resulting in 47 confirmed human cases^[11,23]^. Subsequent outbreaks continued in various African countries, including the DRC, Nigeria, Gabon, Cameroon, Central African Republic, Congo, South Sudan, Sierra Leone, and Liberia, with Nigeria and the DRC experiencing the largest outbreaks between 2017 and 2019^[24–26]^.

### Evolution of mpox nomenclature and global outbreaks

In May 2022, a significant international outbreak of mpox began, prompting the WHO to declare it a global health emergency^[10,11,14]^. This outbreak challenged the traditional understanding of mpox as primarily a zoonotic disease endemic to Africa. In the UK, a traveler from Nigeria with a history of rash entered the country on 3 May 2022, and was confirmed to have mpox on 6 May 2022[27]. By 12 July 2023, the ongoing outbreak, driven by Clade IIb, had reached 112 countries across all WHO regions, with 88 288 laboratory-confirmed cases and 149 deaths reported[3].

On 28 November 2022, the WHO recommended adopting the term “mpox” instead of “monkeypox” to mitigate potential stigma and discrimination associated with the original name^[11,28,29]^. Both “mpox” and “monkeypox” were to be used concurrently for one year, after which “monkeypox” would be officially phased out[11]. Additionally, to further reduce geographic stigmatization, the Central African and West African clades were recommended to be renamed as Clade I (CA) and Clades II and IIb (WA)[17]. This renaming strategy aims to promote a more neutral and less regionally associated terminology in the ongoing efforts to manage and communicate about the mpox outbreak effectively.

In 2024, epidemiological data indicated significant outbreaks in sub-Saharan Africa, particularly in the DRC, which reported over 7000 suspected cases with a case fatality ratio of 5.3%. Children aged 15 years and younger represented 67% of cases and 84% of deaths. Two distinct outbreak patterns were observed: one among adults, notably affecting sex workers, and another primarily affecting children in previously unaffected provinces. A new Clade I MPXV sublineage was identified in South Kivu, with evidence suggesting multiple zoonotic spillover events. Sexual transmission of Clade I MPXV was first documented in April 2023. Concurrently, 27 countries continued to report new Clade IIb cases, including an outbreak among men who have sex with men (MSM) in South Africa. A severe form of Clade II infection has been associated with immunocompromised individuals. These findings indicate evolving transmission dynamics and geographical spread of mpox, necessitating enhanced surveillance and control measures[30].

## Viral entry and pathogenesis of MPXVs

The MPXV life cycle comprises stages such as attachment, entry, and uncoating of the viral core, DNA replication, virion assembly, and release. MPXV infection begins with the virus attaching to the host cell surface. After attachment, virions gain entry via direct plasma membrane fusion or an acidic endosomal pathway, releasing the viral core into the cytoplasm[31]. Several host-specific factors, including potential interactions with extracellular matrix components such as heparan sulfate, glycosaminoglycans, and chondroitin sulfate, have been identified to influence viral entry into host cells. However, the specific receptors for MPXV entry remain unknown, which is noted as an outstanding question in the field[32]. Some studies have also revealed that Poxviruses can infect cells without specific receptors, contributing to their broad host cell susceptibility[33].

**Figure 2.**
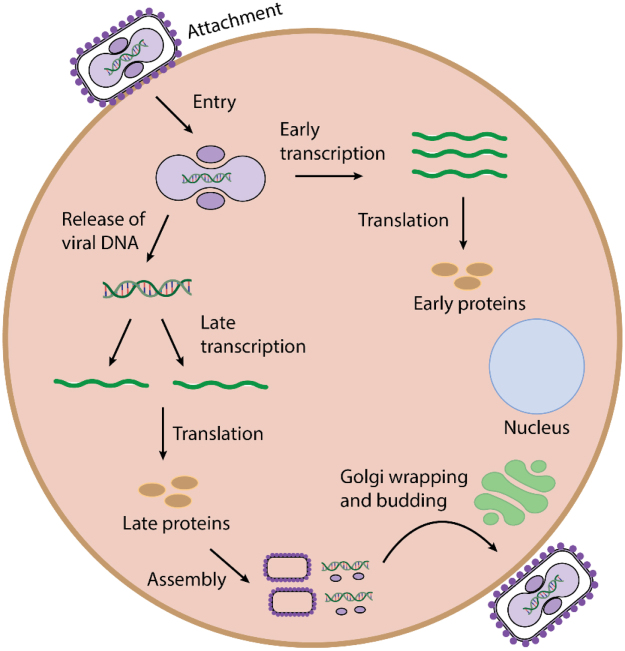
Schematic illustration of viral entry and pathogenesis of mpox virus. The figure illustrates the pathogenesis of the mpox virus from cellular entry to host DNA integration, transcription, translation, and virion protein assembly. The figure further illustrates the process of viral exit following host cell invasion.

Unlike most DNA viruses, MPXV replicates in the cytoplasm of host cells (Fig. 2). In the cytoplasm, replication is initiated by virally encoded DNA-dependent RNA polymerase (RNAP), followed by the early, intermediate, and late stages of protein translation on host ribosomes. Late proteins eventually assemble into IMVs[31]. Some IMVs are wrapped by a double membrane during post-translational modification by the endoplasmic reticulum and/ or Golgi to form an intracellular enveloped virus (IEV)[31]. Studies have revealed that cellular proteins, such as conserved oligomeric Golgi (COG4 and COG7) and vacuolar protein sorting factors (VPS52 and VPS54), are essential in this process^[34,35]^. IEVs fuse with plasma membranes and get released as EEVs. IMVs are released during cell lysis and facilitate local infection, while EEVs, which have an additional membrane layer, enable systemic dissemination[31]. MPXV exhibits broad tissue tropism and can disseminate through lymphatic and blood vessels to infect multiple organs, including the eyes, lungs, liver, kidneys, and genital areas. Furthermore, they can infect various cell types, including Langerhans cells, macrophages, and dendritic cells[32].

The complex interplay between MPXV and host cell proteins highlights critical dependencies that may serve as potential targets for therapeutic intervention. For instance, host proteins such as COG4 and VPS52 are vital for viral morphogenesis and replication, but the precise mechanisms underlying their roles in MPXV pathogenesis remain under investigation[31]. Furthermore, two MPXV genes, A6R and E8L, have been identified as potential drug targets important for viral replication. The E8L gene is thought to be involved in cell attachment, similar to its vaccinia virus ortholog D8L. Inhibiting E8L significantly decreases MPXV replication, suggesting that it may play a role in viral entry. The A6R gene is essential for MPXV replication and likely plays a critical role in viral gene expression and replication, as its vaccinia ortholog A5R functions as a precursor for RNA polymerase[36].

## Genetic determinants of MPXV transmissibility

Insights into the genes of MPXV are crucial for deciphering its pathogenesis, interactions, and potential for transmission. The distinct genetic make-up of MPXVs from other orthopoxviruses contains a combination of conserved and renounced genes that play essential roles in their evolutionary trajectory and interaction with host species[37].

Notably, studies have identified distinctions in the D14R gene across MPXV lineages. This gene codes for the MOPICE protein, which is an inhibitor of complement enzymes. Clade I MPXV possesses full-length genes, while Clade II MPXV possesses a 10-kbp deletion, which reduces the presence of MOPICE in greater quantity[37]. While some studies have demonstrated that the removal of MOPICE reduced mortality and morbidity in prairie dog and non-human primate models, the insertion of the gene into Clade IIa did not increase the severity of disease^[38,39]^. Genomic surveillance of Clade Ib has shown truncations of MOPICE. Since Clades Ib and IIb exhibit higher transmission rates in humans compared to Clades Ia and IIa, it is believed that MOPICE may play a crucial role in host-disease susceptibility by reducing the transmission potential. This could be an adaptation that favors human-to-human spread by minimizing lesions and scarring, thereby promoting undetected spread[37].

Another interesting evolutionary mechanism that may be responsible for increasing MPXV fitness toward human infection and transmission is the APOBEC3 mutation. This mutation is a cytolytic mRNA-editing enzyme that changes guanine (G) to adenine (A) and cytosine (C) to thymine (T). The 2022–2023 outbreak, which comprised mainly Clade IIb, lineage B.1, and the descendants, showed more single-nucleotide changes than expected for an orthopoxvirus. These were roughly dozens of SNPs compared with the pre-2022 strains[40]. The excess in these SNPs characterized by G–A/C–T changes may indicate host-mediated editing, especially by the APOBEC3 enzyme, compared to random drifts. Significantly, the APOBEC3 enzymatic host editing mediations have continuously changed, demonstrating microevolution patterns and adaptations[41]. Genomic analyses show APOBEC3 mutations producing higher than expected numbers of non-synonymous and nonsense mutations across the genome, which follows the notion of reductive evolution of orthopoxviruses. These changes could alter the viral transmissibility, tissue tropism, and diagnostic targets. The mutations highlight the need for continued surveillance, as any of them could deactivate genes associated with murine host interaction and eventually generate a better human-adapted virus[37]. Several APOBEC3 mutations, such as OPG210 (surface glycoprotein and T-cell suppressor), OPG047 (BTB-kelch domain contributor of virulence and lesion size), and many more have been observed in genes of Clade IIb MPXV. Although these mutations have not been proven to be responsible for increased human-to-human transmission, studies have identified codon biases preferentially used in human hosts[37]. Therefore, there is a need for further studies to identify the nature of these mutations and their contribution to MPXV virulence. Additionally, scaling up sequencing programs in endemic and non-endemic settings, especially with epidemiological metadata, is necessary[40].

## Phylogenetic analysis of mpox clades

**Figure 3.**
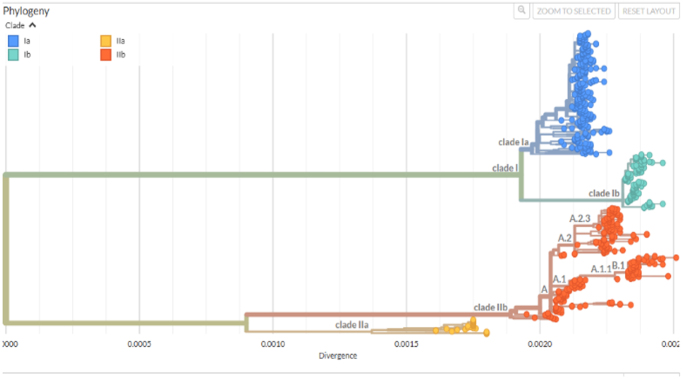
Current phylogenetic tree of mpox viruses (Source: image obtained from Nextstrain). The figure illustrates the evolutionary divergence of the two major mpox clades: I and II into subclades Ia, Ib, IIa, and IIb.

MPXV began its independent evolution approximately 3500 years ago, with the West African subtype emerging about 600 years ago[15]. Current phylogenetic analysis of MPXVs across all clades with 550 genomes deposited at Nextrain reveals two primary lineages, Clade I and Clade II, with each primary clade being further divided into two subclades: Clades Ia and Ib, and Clades IIa and IIb, respectively (Fig. 3)[42]. Within Clade IIb, several lineages have been identified, including A.1, A.1.1, A.2, and B.1. The B.1 lineage has been predominantly responsible for the global mpox outbreak observed since 2022[43]. Further phylogenomic reconstruction of MPXV Clade IIb B.1 using 1777 whole genomes from 37 countries revealed several small, mostly independent clusters with geographic consistency[44]. The phylogenetic tree showed localized clusters appearing as evolutionary blind lineages with few or no descendants[44]. Branch lengths in the phylogenetic tree suggest low diversification among the samples[44]. A maximum likelihood phylogenetic tree revealed the emergence of new clusters, designated as sub-lineages B.1.1 to B.1.14, within Clade IIb[45]. The B.1 lineage has been distinguished by a single nucleotide change in the OPG-098 gene at position 77383G>A (E162K)[45]. Sub-lineages such as B.1.1, B.1.2, and B.1.6 have the most sequenced genomes[45]. All documented sequences with a collection date in 2022 belong to MPXV Clade IIb, mainly lineage B.1, followed by lineage A.2, except four Clade I sequences from the Democratic Republic of the Congo[46]. The 2022 outbreak sequences belonging to Clade IIb clustered with the 2018 and 2019 cases in the UK, which were linked to travel from Nigeria[46]. Depending on the analytic method, such as whole-genome versus partial-gene sequencing, these strains may cluster differently, revealing phylogenetic ambiguity in the evolutionary transition zones. Understanding the phylogenetic relationships among mpox virus clades can help with tracking the virus’s evolution, transmission patterns, and inform public health interventions.

## Geographical distribution: Clade IIb vs. other clades

### Clade IIb

Clade IIb is mainly found in the DRC and potentially in other regions of Central Africa^[1,47]^. Being the primary driver of the 2022 global outbreak[48], it has been additionally identified in over 100 countries, with significant clusters in Europe (including the UK, Spain, and Germany), North America (such as the USA and Canada), and parts of South America, like Brazil[49]. Unlike earlier clades that were more rural, Clade IIb has shown a strong presence in urban areas, particularly among MSM communities[50]. Its enhanced capacity for human-to-human transmission, especially within closely connected social and sexual networks, has driven its rapid spread across various regions[51]. Furthermore, while it shares some clinical similarities with Clade I, data on this clade remain limited, making its epidemiological characteristics less clear. Clade IIb’s overlap with Clade I complicates efforts to study its distinct transmission patterns.

The widespread nature of Clade IIb, particularly in non-endemic regions, underscores the importance of establishing enhanced global surveillance networks to monitor and respond promptly to emerging cases[52]. These geographical differences can also impact vaccine effectiveness, as regional differences in clade prevalence may affect how vaccines perform across diverse populations[53]. This variation necessitates tailored vaccine strategies that account for the specific clades present in different areas. Furthermore, the geographical shift of mpox requires healthcare professionals in newly affected regions to develop specialized skills in diagnosing and managing cases, particularly those associated with the unique transmission patterns of Clade IIb[54].

### Endemic (Clades Ia, Ib, and IIa)

Contrary to the widespread nature of Clade IIb, other mpox clades maintain more regionalized distributions, remaining largely within their endemic zones. Clade Ia remains predominantly confined to the Congo Basin, affecting countries such as the DRC and the Republic of Congo[26]. It is known for its higher mortality rates and severe clinical symptoms. The transmission remains largely zoonotic, with limited instances of human-to-human spread.

**Figure 4.**
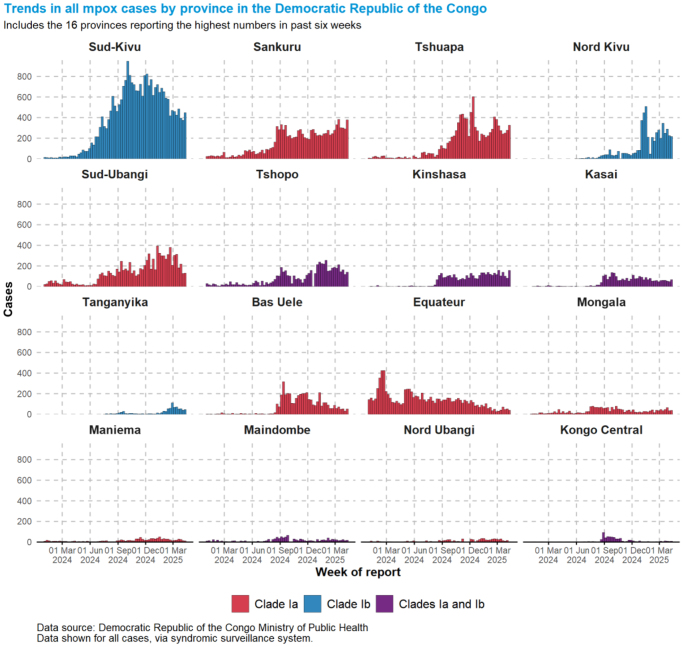
Showing the trends of Mpox clades in DRC according to provinces (World Health Organization, 2025). This figure is an illustration of the mpox cases characterized by their clades in different DRC provinces between 2024 and 2025.

Clade Ib has been predominant in multiple provinces within the DRC, such as Sud-Kivu, Kasai, Mai-Ndombe, and Lomami. Its prevalence in Lomami has been minimal, with less than 50 cases identified, as demonstrated in Figure 4. However, Sud-Kivu province has experienced the highest cases between March 2024 and March 2025, with over 800 cases recorded around November and December 2024[55].

Clade IIa is primarily associated with West African nations like Nigeria, Ghana, and Sierra Leone[56], and Clade IIa has historically shown lower mortality rates and reduced human-to-human transmission compared to Clade Ia[57]. However, recent outbreaks in Nigeria between 2017 and 2019 revealed its potential for larger-scale human-to-human transmission[58]. The varying geographical distribution of mpox clades carries important consequences for public health approaches and future research endeavors. One major implication is the need for robust surveillance systems.

## Analysis of clinical presentation across mpox clades’ infection

Mpox infection is associated with typical symptoms, including rash, fever, sore throat, headache, muscle aches, back pain, low energy, and swollen lymph nodes. However, the course and severity of the clinical presentation may vary with different clades, routes of transmission, host susceptibility, and amount of inoculated virus (Table 1).

**Table 1:** Summary table of clinical and epidemiological characteristics across mpox clades

Characteristics	Clade Ia	Clade Ib	Clade IIa	Clade IIb (Lineage A)	Clade IIb (Lineage B.1)	References
Transmission	Zoonotic (60–75%); some human-to-human (H2H) spread (40–35%)	99% H2H spread	100% zoonotic spread	10% zoonotic; 30% H2H; 60% unknown	99% H2H, often sexual transmission	^[59–62]^
Age and sex	90% < 15 years; no male predominance	Predominantly adults (85%); mostly men (>98%)	70% adults; no significant sex difference	80% adults; 70% male predominance	>99% adults; > 98% male predominance, especially in MSM communities	^[63,64]^
Primary lesion sites	Predominantly face	Anogenital (70–87%); some oral and genital lesions (40–60%)	Site of animal contact, especially hands or arms	Mixed (often face and extremities)	Predominantly anogenital (localized)	^[65,66]^
Lymphadenopathy	80%; submaxillary and cervical	42% (site often inguinal)	70%; cervical region	70%; mixed cervical and inguinal	50%; primarily inguinal	^[64]^
Systemic symptoms	High fever (80%); chills; myalgia	Fever (60%), chills, anorectal pain	Moderate fever and fatigue (73%)	Mild systemic symptoms in most cases (57%)	42–57%; significant systemic symptoms in HIV+ patients	^[63,67]^
Complications	Rare encephalitis; secondary infections	Proctitis, anorectal pain, and severe genital complications	Rare; complications generally mild	Localized lesions; infrequent complications	Severe anorectal and genital pain in immunocompromised patients	^[68,69]^
Mortality	5–10%; higher in children and immunocompromised individuals	<1%; lower than Clade Ia	0%	3–5%; highly dependent on patient comorbidities	0.19%; very low in healthy individuals	^[64,70]^
Impact of HIV	Limited; no significant correlation noted	High prevalence in HIV+ patients; anorectal pain and severe genital disease observed	Limited data	High impact on presentation in HIV+ patients, with more systemic disease	Significant complications and prolonged recovery in HIV+ patients	^[67,71]^
Unique features	Cases of intrauterine transmission, especially in stillbirths	Sexual transmission established; proctitis and anogenital lesions are common	Predominantly zoonotic; localized lesions at the site of contact	Mixed presentations; concurrent lesions at different stages	New presentations such as mpox whitlow (digital lesions)	^[59,70,72]^

The table demonstrates the comparative characteristics of the different mpox clades, highlighting the clinical and epidemiological features. Clade Ia involves both zoonotic and human-to-human transmission, with no male predominance and a relatively high mortality rate (5–10%). Clade Ib is primarily spread through human-to-human contact, occurs mostly in men, and has a mortality rate of less than 1%. Clade IIa also spreads via human-to-human transmission, shows no significant sex differences, and has no recorded mortality. Clade IIb involves both zoonotic and human-to-human spread, shows a male predominance, and has variable mortality: approximately 3–5% in lineage A and 0.19% in lineage B.1.

### Comparative analysis of skin lesions

As a newly identified clade, Clade Ib is still under investigation, with researchers working to better understand its unique characteristics and clinical implications. Nevertheless, the emergence of Clade IIb mpox virus has brought significant changes to the clinical presentation of the disease, particularly in terms of skin lesions. While endemic infections typically present with hundreds to thousands of lesions distributed globally across the body, Clade IIb infections often manifest with 10 or fewer lesions, sometimes even presenting as solitary lesions[73]. This stark contrast in lesion count is further emphasized by a study showing that 89% of Clade IIb patients had 25 or fewer lesions[74].

The distribution of lesions also differs markedly between clades. Endemic infections tend to have a global distribution, whereas Clade IIb infections are often localized around the site of inoculation[73]. This localization is particularly evident in the anogenital area, which was the most frequently affected body site in 76.6% of Clade IIb cases[74]. This pattern differs significantly from the centrifugal distribution (more pronounced in extremities and the face) observed in previous outbreaks[75].

Another distinguishing feature is the stage of lesion evolution. In endemic infections, lesions are usually at the same stage of evolution, presenting a monomorphic appearance. Conversely, Clade IIb infections may exhibit lesions at different stages of evolution[73], often resulting in a polymorphic rash[75]. The typical progression of lesions in Clade IIb infections doesn’t always follow the classic stages seen in past outbreaks (macules to papules to vesicles to pustules to umbilicated pustules to crusts)[75].

Histologically, mpox lesions progress through five distinct stages: macule, papule, pustule, early ulceration, and late ulceration. Each stage has unique features, ranging from subtle changes in the early macular stage to more pronounced changes in later stages, including epidermal pallor, ballooning of keratinocytes, multinucleation, and eosinophilic intracytoplasmic inclusion bodies (Guarnieri bodies)[76]. The lesions typically appear as edematous whitish papules with marked umbilication and central crusting (pseudo pustules), evolving into crusts and erosions, with some resolving as superficial atrophic scars[77]. Notably, lesions on palms or soles, which were common in prior outbreaks, are less common in Clade IIb infections[75]. Additionally, Clade IIb infections show strong involvement of hair follicle epithelia and sebaceous glands, which may play a role in the pathogenesis and distribution of lesions^[76,77]^.

### Systemic symptoms and disease progression

The systemic symptoms and disease progression of Clade IIb Mpox infections show notable differences compared to endemic infections. In endemic cases, prodromal symptoms such as fever, fatigue, and headache are frequently present[73]. However, in Clade IIb infections, these symptoms are only occasionally present, with 74.8% of patients reporting systemic symptoms[74]. Specifically, 67% experienced fatigue, 57% reported fever, and 18% did not present with any prodromal symptoms[75]. This contrasts with past outbreaks where fever and systemic symptoms were more prominent[75]. Lymphadenopathy presents another point of divergence. Endemic infections typically feature prominent and painful lymphadenopathy[73]. In Clade IIb infections, lymphadenopathy may or may not be present, with one study reporting its occurrence in 46.5% of patients[74].

The incubation period for Clade IIb infections is shorter (6–11 days) compared to endemic infections (7–17 days)[73]. Despite these differences, the majority of Clade IIb cases were mild, with low impact on daily activities. However, 22.6% of patients in one study had severe disease, defined by criteria including >100 skin lesions, complications, severe proctitis, or hospitalization[74]. The disease progression in Clade IIb infections appears to follow a staged immunological response characterized by an initial T-cell response dominated by CD4+ T-helper cells, followed by a cytotoxic CD8+ T-cell response that leads to keratinocyte degeneration and necrosis. This is succeeded by the recruitment of neutrophil granulocytes in later stages, with massive neutrophil diapedesis leading to vascular closure and ulceration in late stages[76].

### Atypical presentations and diagnostic challenges

The emergence of Clade IIb mpox has introduced several atypical presentations and diagnostic challenges. One of the most striking differences is the occurrence of disease without rash. While this was not reported in endemic infections, Clade IIb infections have shown cases of rectal, pharyngeal, or mucosal lesions without rash[73]. In one study, 6.5% of patients presented without skin lesions, instead showing only mucosal involvement (proctitis, urethritis, and tonsillitis) or systemic symptoms[74].

The early macular stage of Clade IIb mpox can be easily missed or misdiagnosed histologically, as it lacks characteristic features and can resemble other conditions like drug reactions, cutaneous lupus erythematosus, pityriasis lichenoides, or cutaneous T-cell lymphoma[74]. Differentiation from herpes virus infections can be challenging, especially in advanced stages, due to similar epidermal changes[74]. Some patients with Clade IIb infections have presented with genital ulcers that were painless with bilateral inguinal lymphadenopathy, mimicking primary syphilis. In other cases, proctitis was the primary presentation, with no visible rash in one-fifth of these cases[75]. The location of lesions in Clade IIb infections often correlates with the type of sexual activity performed, which was not typically seen in past outbreaks[75]. Complications in Clade IIb infections have included anal pain, cellulitis, urinary signs, ocular infections, abscess, lymphangitis, and paronychia[73]. This differs from endemic infections, which often lead to complications such as dehydration, diarrhea, bronchopneumonia, ocular infections, and encephalitis[73].

Due to these atypical presentations, maintaining a high index of suspicion and a low threshold for testing in at-risk individuals is crucial, even in the absence of typical skin lesions[74]. The presence of lymphadenopathy, proctitis, or skin lesions in sexually active MSM should raise suspicion for mpox[74]. Diagnostic tools such as immunohistochemical staining with anti-Vaccinia virus antibodies can be useful for differentiation, but they are negative in the early macular stage[74]. PCR testing is crucial for accurate diagnosis, especially in the early stages when histological changes are subtle[74]. Electron microscopy, when available, can contribute to confirming the diagnosis by demonstrating numerous viral particles of mpox in affected keratinocytes[77].

## Diagnosis of clade MPXVs

### Molecular diagnostic techniques: PCR and gene targets

Real-time polymerase chain reaction (RT-PCR) remains the primary and most reliable method for confirming MPXV infection[78]. RT-PCR is typically performed on both pharyngeal and vesico-pustular fluid swabs, with the RTPCR018-LPD-R kit from Vircell Microbiologists being one example of a commercially available test[78].

A new diagnostic MPXV real-time PCR detection kit (Sansure MPXV Nucleic Acid Diagnostic Kit) has shown promising results. This assay demonstrated high analytical sensitivity, with a limit of detection (LoD) of less than 200 copies/mL for whole blood samples, 165 copies/mL for vesicle samples, and 119 copies/mL for pustule samples. The kit exhibited 100% sensitivity in various sample matrices over 21 days and 100% specificity against 19 potentially cross-reacting pathogens. In clinical performance testing, it demonstrated a diagnostic sensitivity of 100.00% and a specificity of 96.97%, outperforming a CE-certified comparator kit by a slight margin[79].

Another study evaluated a laboratory-developed test using BioGX Xfree hMPXV/OPXV reagents for detecting non-variola Orthopoxviruses and Mpox virus DNA. This test utilized two primer/probe sets: G2R_G, which is specific to all known MPXV strains, and E9L-NVAR, which is capable of detecting various non-variola orthopoxviruses. The assay demonstrated high sensitivity (100%) and specificity (100%) with an LoD of 500 copies/mL on the BD MAX™ System and 250 copies/mL on the pixl.16 real-time PCR platform[80]. In cases where PCR is unavailable or the diagnosis is uncertain, skin biopsy for histopathological examination and transmission electron microscopy can serve as valuable diagnostic aids[78].

### Serological testing: applications and limitations

Serological testing for mpox faces several challenges, primarily due to the cross-reactivity between antibodies against MPXV and other orthopoxviruses such as cowpox virus and vaccinia virus. This cross-reactivity allows the use of less pathogenic orthopoxviruses as antigens in serological assays for MPXV antibody detection[81].

Several serological methods have been evaluated, including the immunofluorescence assay (IFA), which can detect both IgG and IgM antibodies against MPXV, with comparable titers for most samples using different orthopoxvirus species[81]. The neutralization test detects neutralizing antibodies against MPXV, revealing larger differences in titers when using different orthopoxviruses compared to IFAs[81]. An optimized enzyme-linked immunosorbent assay (ELISA) protocol has been developed for higher throughput testing, capable of detecting both IgG and IgM antibodies, and ELISA results showed good correlation with IFA titers, especially for samples with higher titers[81]. Additionally, a multiplex assay (MesoScale Diagnostics – MSD) testing multiple antigens demonstrated the best overall performance among several commercial serological tests evaluated[82].

However, these serological tests have limitations. They may not easily distinguish between vaccination and infection due to cross-reactivity[81]. Additionally, none of the assays could reliably differentiate between mpox-positive and vaccinated samples[82]. The E8L antigen consistently performed best across different assays in distinguishing mpox-positive samples from negatives[82]. Despite these limitations, serological methods have important applications. They can aid in diagnostics, are key for epidemiological studies, and can help in detecting acute infections due to their higher dynamic range and ability to detect IgM antibodies[81]. Serology also has potential for evaluating population-level exposure and immunity to mpox, identifying asymptomatic or subclinical cases, and assessing vaccine interventions[82].

### Emerging point-of-care diagnostics

While not explicitly labeled as a point-of-care diagnostic, the pixl.16 real-time PCR platform shows potential in this direction. It’s described as compact and portable, offers an extraction-free workflow, provides results in less than 60 min, and can test up to 16 samples at once. These characteristics suggest that it could be used in settings where rapid, on-site testing is needed, potentially moving Mpox diagnostics closer to point-of-care applications[80]. Other point-of-care diagnostics include the Xpert mpox, the Cobas MPXV assay, the Ustar EasyNat platform, and the QIAGEN QIAstat-Dx. Emerging technologies with diagnostic potential include the Alpha fold, CRISPR-based assays, and Isothermal Amplification techniques, such as the recombinase polymerase amplification and loop-mediated isothermal amplification.

To ensure biosafety in handling potentially viremic samples, a virus inactivation protocol has been developed. This protocol achieved at least a five-log-level depletion of infectious poxvirus particles, allowing safe handling of samples in BSL-2 laboratories[81]. While PCR remains the gold standard for mpox diagnosis, serological tests are improving and have important epidemiological applications. The development of faster, more portable PCR platforms may pave the way for true point-of-care diagnostics in the future.

## Treatment approaches for MPXVs

### Current antiviral therapies and their efficacy

Tecovirimat (TPOXX) is considered the primary antiviral for severe mpox cases and can be administered orally or intravenously^[83,84]^. It inhibits the viral protein p37, which is involved in virus maturation and spread[84]. A study on 255 patients revealed a median interval of 3 days to first subjective improvement with limited adverse events[83]. Animal studies have demonstrated improved survival rates in non-human primates and rabbits infected with lethal doses of MPXV or rabbitpox virus[84]. Limited observational data in humans suggest potential efficacy, with one patient becoming PCR negative 48 hours after starting treatment, while two patients with severe proctitis reported improvement within 48 hours. Additionally, a case series of 25 patients showed 40% lesion resolution by day 7 of therapy and 92% by day 21[84]. However, resistance has been detected in a small number of patients with advanced HIV[83].

Brincidofovir and Cidofovir can be added to tecovirimat treatment for severe cases, usually administered once weekly for 2 weeks[83]. Brincidofovir is FDA-approved for smallpox treatment and has shown efficacy in animal models against orthopoxviruses, but it has more side effects than tecovirimat, including diarrhea, nausea, and hepatotoxicity^[84,85]^. Cidofovir exhibits activity against orthopoxviruses; however, its use is limited by IV administration and nephrotoxicity^[84,85]^. Both have FDA black box warnings and require close monitoring[83].

Trifluridine is used for ocular mpox infections and has been shown to inhibit replication of several viruses, including vaccinia virus. It has demonstrated efficacy against ocular vaccinia virus infections in animal models and humans[83]. There is still a significant gap concerning mpox treatment, especially targeting the specific clades.

### Immunotherapy: potential and challenges

Vaccinia immune globulin intravenous (VIGIV) provides passive immunoglobulin G antibodies against vaccinia virus, which might offer cross-protection against mpox^[83,85]^. It is recommended for patients unable to mount a sufficiently robust immune response, such as those with HIV-related CD4 count <350 or after solid organ transplantation[83]. VIGIV is FDA-authorized for treating complications from vaccinia vaccination, but it has limited evidence on efficacy against mpox and smallpox[85]. Its safety profile is believed to be favorable, but caution is advised in patients with ocular mpox involving the cornea[83]. VIGIV is available in limited supply and must be administered under an IND application^[83,85]^.

Recent research has identified several human monoclonal antibodies (mAbs) that showed cross-neutralizing activity against multiple orthopoxviruses, including MPXVs[86]. A mixture of 4–6 monoclonal antibodies targeting different viral proteins (both mature virion and enveloped virion forms) showed superior neutralizing and protective effects compared to individual antibodies or vaccinia immune globulin^[86,87]^. Key antibody targets identified included proteins A33, B5, A27, and L1[86]. The antibody mixture designated MIX6 provided protection in mouse models of both respiratory and systemic orthopoxvirus infection when given prophylactically[86]. These findings suggest that human mAbs could potentially replace VIG for the treatment of orthopoxvirus infections, including mpox^[86,87]^.

### Emerging targeted therapies and clinical trials

The development of new targeted therapies and clinical trials for mpox treatment is an active area of research. The NIH-funded Study of Tecovirimat for Human Mpox (STOMP) trial is an ongoing clinical trial evaluating the effectiveness of tecovirimat[83]. Additionally, the CDC has partnered with NIH to study the role of immune dysregulation in severely immunocompromised HIV patients started on antiretrovirals through the Virologic and Immunologic Characteristics of Severe mpox Among Persons with Advanced HIV (VIRISMAP) study[83].

Researchers are exploring mRNA and multi-epitope vaccines as potential new treatment approaches. Ten MPXV proteins have been identified as potential vaccine targets, with epitopes mapped for B cells, CTLs, and helper T lymphocytes[85]. Molecular docking and immune simulation studies have demonstrated the potential efficacy of these vaccine candidates[85].

Several compounds from natural sources are being investigated for their potential antiviral properties against mpox. Four compounds from the African natural compound database have been identified as potential disruptors of MPXV immune evasion mechanisms by targeting the F3L-dsRNA interaction[85]. Additionally, four compounds from Traditional Chinese Medicine (TCM27763, TCM33057, TCM34450, and TCM31564) have been identified as potential inhibitors of MPXV I7L protease, showing superior pharmacological potential compared to the control[85].

Thymidylate kinase (TMPK) inhibitors are another area of interest. Several compounds from various databases (TCM, South African natural, Natural Product Activity and Species Source, Collection of Open Natural Products) have been identified as potential TMPK inhibitors, demonstrating significant pharmacological activity against TMPK[85].

The development of resistance to tecovirimat is a potential concern that requires further study and monitoring[84]. There is a pressing need for more robust clinical trials to fully evaluate the efficacy of tecovirimat and other antivirals for MPXV infection in humans, particularly for special populations like children and pregnant women[84]. Controlled studies focused on understanding the impact of monotherapy or combination therapy on virus shedding, duration of illness, and clinical outcomes, particularly for patients with severe immunocompromise, are also needed[83].

## Vaccines against mpox: VARV and new vaccine development

The recent outbreak of mpox has highlighted an urgent need for robust vaccination strategies to curb the spread of this zoonotic virus. While the vaccinia virus (VARV) vaccine, originally developed for smallpox, has shown cross-protective efficacy against mpox, the virus’s evolving nature and the emergence of novel clades underscore the necessity for ongoing research and innovation in vaccine development[50]. Other vaccine formulations developed with potential against mpox include mRNA vaccines[88]. The effectiveness of existing vaccines is increasingly challenged by the virus’s capacity for mutation, necessitating a proactive approach to vaccine strategy that incorporates genomic insights and community-specific considerations.

Historically, the VARV vaccine played a crucial role in the global eradication of smallpox, with documented cross-protective effects against mpox facilitating its use in outbreak scenarios[89]. Research indicates that this vaccine provides approximately 85% protection against mpox infection[90]. Nevertheless, there are limitations, including the potential for severe side effects in immunocompromised individuals, which limit its applicability in certain populations[91]. Additionally, traditional production methods that rely on live animals may be insufficient to meet the global demand for vaccines, necessitating a re-evaluation of production practices and an emphasis on innovative methods to ensure availability[92]. The duration of protection remains under investigation, particularly regarding different age groups and mpox clades[93].

Innovations in vaccine development are gaining momentum, with the Modified Vaccinia Ankara (MVA) vaccine emerging as a promising candidate due to its improved safety profile[94]. MVA has demonstrated efficacy in both animal and human trials, exhibiting fewer side effects compared to traditional VARV vaccines[95]. Approved for use in various regions, including the USA and several EU member states[96], MVA represents a significant advancement in the fight against mpox. Other candidates, such as the LC16 vaccine developed in Japan, show a favorable safety profile and are currently under evaluation for broader use^[97–99]^. Additionally, research into subunit and recombinant vaccines, which target specific mpox antigens, promises a safer approach, especially for immunocompromised individuals^[100,101]^, with several candidates progressing through preclinical and early clinical development stages[102]. However, the distribution of vaccines faces significant inequities, particularly in low-income countries where mpox is endemic, further complicating global efforts to control the disease[103]. Vaccine hesitancy, fueled by misinformation during the COVID-19 pandemic, poses additional challenges to achieving high vaccination rates[104], emphasizing the need for robust health communication strategies[105]. As these innovations unfold, addressing these challenges will be crucial to ensuring effective vaccination efforts in the face of evolving threats. Multiple vaccines, such as the ACAM2000 and MVA-BN (Jynneos), have been approved against mpox, supplementing the VARV smallpox vaccine. However, the supply of these vaccines remains limited due to the limited manufacturing capacity, especially in low- and middle-income countries. There is also limited information concerning their efficacy in various populations, warranting more molecular studies and trials.

## Public health response, challenges, and global health implications

### Public health response: local and international efforts

Over time, mpox mitigation strategies have evolved dynamically, with improvements in diagnostics and testing. There wasn’t a public health response strategy around 1958 when the virus was first discovered among monkeys, mainly because it was still confined among non-primates[106]. Public health strategies were also limited during the 1970s when the first human case was discovered in the DRC, and these prioritized Smallpox surveillance and eradication. However, stringent strategies, such as quarantine, rapid response, and animal surveillance, were implemented in early 2003 following the mpox outbreak in the USA^[106,107]^. Other robust public health strategies implemented globally include enhanced surveillance, international collaboration, enhanced funding for mitigation, mass vaccination, the One Health approach, and public health education. A rapid response team established by the UK health authorities in 2022 coordinated effective and extensive contact tracing of individuals infected with or confirmed to have mpox among the population[40]. Individuals confirmed to have the mpox infection are also monitored for a period of three weeks through their effective surveillance system. Additionally, individuals with a higher risk of contact with infected individuals are being vaccinated as a preventive measure. If these public health measures are established in an addendum to vaccination, greater outcomes will be achieved. Previous studies have demonstrated the importance of vaccination with smallpox vaccines as a public health response; however, mass vaccination has been disproved by the WHO to be needed in curtailing the mpox outbreak. Interestingly, the adoption of ring vaccination may be effective in containing the mpox outbreak among risk groups and their close contacts^[12,24]^. Sharing of vaccines with other affected countries with limited vaccine supply has been recommended by the WHO in achieving global control of the mpox outbreak[18].

### Challenges to prevention and control

Mpox remains a significant zoonotic disease of public health concern, and as such, it requires national and international responses in affected countries. Adopting a multidisciplinary One Health approach can help minimize the occurrence of outbreaks. The COVID-19 wave prompted many African countries to enhance their laboratory diagnosis capabilities; as such, all African countries have PCR machines to test for mpox, although this may not be sufficient. While PCR machines are available in all African countries, the sequencing of the mpox genes can only be done in seven African countries[19]. Additionally, the lack of reagents and training on specimen collection, handling, and testing poses a significant challenge to the prevention and control of mpox in African countries (Fig. 3). Specimen viability is rarely achievable in some places due to a lack of refrigerators and reliable power supply in healthcare facilities where specimens are collected before being transported to test centers. Additionally, poor road networks, as well as a lack of techniques and supplies for infection prevention and control, are a hindrance to achieving the prevention and control of mpox in rural health facilities of African countries^[19,20,46]^.

However, some diagnostics, vaccines, therapeutics, and effective surveillance system tools required to monitor and prevent mpox are not accessible to most African countries[76]. As much as smallpox vaccines are proven antidotes for controlling and preventing mpox, inherent challenges are barriers to achieving an mpox-free global space. The major vehicle of these vaccine-related challenges is traceable to inequities among developed and developing or underdeveloped countries[12]. Geographical location, poor transportation, education, racism, language barrier, vaccine failure, and poor government policies are some of the challenges of using vaccines as a public health response to mpox[21]. To achieve maximum prevention and control of mpox, African countries should address the challenge of healthcare worker shortages. The lack of enough human resources in the healthcare sector deters the process of sample collection for laboratory examination of suspected cases. Additionally, most of the few available healthcare providers in African countries do not possess the proper knowledge, skills, and experience required for effective diagnosis and identification of mpox.

**Figure 5.**
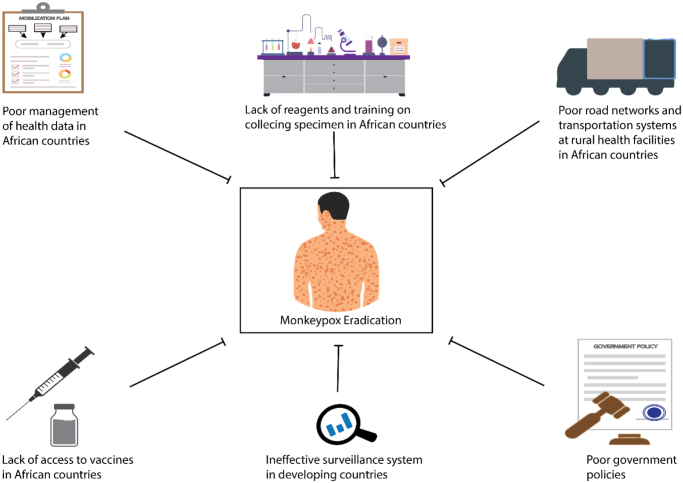
Challenges of mpox eradication. The figure illustrates the different challenges in mpox eradication, divided into six major categories of transportation infrastructure, governance policies, surveillance systems, vaccine products, health data management, and laboratory testing capacity.

Poor data management of routine health data and information is a characteristic of most sub-Saharan African countries (Fig. 5). These data are poorly managed, incomplete, inconsistent, and not readily available[108]; hence, they are rarely used in decision-making in curtailing outbreaks like mpox. Most primary health centers in African countries lack the tools and techniques for case identification and recording. All these delinquencies contribute to delayed detection and case reporting of mpox outbreaks and consequently interfere with the strategies put in place to prevent and control the outbreak and further spread of the outbreak is promoted[22].

Previous outbreaks are characterized by smaller transmission chains; however, the 2022 mpox outbreak may result in a decline in population immunity, leading to epidemics with a reproduction rate greater than 1[25]. Additionally, poor reporting of cases in developing and underdeveloped countries does not give a true picture of the outbreak. It is not impossible to have infected people from this region who are asymptomatic or subclinical but are carriers of the mpox virus^[26,40]^. One of the challenges of previous outbreaks of SARS-CoV and COVID-19 lies in the large population of asymptomatic cases. It is worrisome to think that the 2022 mpox outbreak followed a similar path, and the mpox virus might have found a solution and adapted to efficient human-to-human transmission[26]. The recent findings about asymptomatic carriers of the mpox virus identified in sexual health clinics are evidence of the adaptation of the virus to efficient human transmission[23]. However, there is limited evidence on the infectious level of these carriers, but it is a great concern as it will have an impact on global public health. The virus may mutate or evolve via APOBEC3 actions[109].

The 2022 mpox outbreak has a distinctive epidemiological niche among sexually active individuals who are not immune. Classically, mpox transmission requires direct contact with lesions, placing most people at risk including healthcare workers. Unfortunately, most documented cases in the 2022 mpox outbreak were among MSM with multiple partners. This is evidenced in the high number of positive cases recorded among MSM under 40 years of age, and as such, mpox may soon become an established STD[26]. The pattern of transmission and symptoms of infected cases is a concern, as variation in clinical presentation may affect a physician’s suspicion level in a suspected case[26].

Overall, the 2022 mpox outbreak implies that routine examination for mpox should be compulsory as part of the disease spectrum in patients presented for genitourinary assessment[24]. Additionally, the inclusion of pox virus primers in multiplex PCR assays in rapid and traditional diagnostic laboratories should be prioritized. Vaccination with smallpox vaccines or mpox-specific vaccines for risk groups, such as immunocompromised patients and healthcare providers, should be considered not only as post-infection prophylaxis but also as pre-infection prophylaxis[24].

## Role of genomic surveillance in public health response

Genomic surveillance has become a crucial element in managing infectious disease outbreaks, offering insights into the genetic evolution, transmission dynamics, and potential shifts in pathogen virulence or transmissibility (Table 2)[110]. This method involves systematically analyzing pathogen genomes, enabling health authorities to monitor the spread and adaptation of viruses. In the context of mpox, genomic surveillance proved vital in unraveling the dynamics behind Clade IIb’s rapid emergence during the 2022 global outbreak[40].

**Table 2:** Key roles of genomic surveillance in public health response to mpox

Key role	Description	Impact	Example
Mutation tracking	Genomic surveillance enables the identification and monitoring of viral mutations that may impact the virus’s behavior or clinical presentation	Early detection of mutations associated with increased transmissibility or virulence allows for rapid public health interventions^[40]^	The identification of mutations in the Clade IIb virus that potentially enhanced human-to-human transmission prompted targeted interventions in affected communities^[111]^
Outbreak control	By providing high-resolution data on viral lineages, genomic surveillance facilitates the tracing of infection chains	This information helps public health authorities implement more effective contact tracing and targeted containment strategies^[112]^	During the 2022 outbreak, genomic data helped identify transmission clusters within specific social networks, enabling focused interventions^[113]^
Development of diagnostics	Genomic information is crucial for the development and refinement of diagnostic tests	It ensures that PCR and serological tests remain effective as the virus evolves^[114]^	The rapid sharing of Clade IIb genomic sequences allowed for the quick adaptation of existing diagnostic protocols to detect the new variant^[115]^
Vaccine development and efficacy monitoring	Genomic surveillance informs the development and ongoing assessment of vaccines	It helps ensure that vaccines remain effective against circulating strains and guides the development of new vaccines if necessary^[89]^	Continuous monitoring of Mpox genomic data is essential for evaluating the long-term efficacy of existing smallpox vaccines against evolving Mpox strains^[11]^
Prevention of future outbreaks	By monitoring both human and animal populations, genomic surveillance can detect potential zoonotic spillover events early	Early detection of novel variants or increased viral activity in animal reservoirs can trigger preemptive public health measures^[116]^	Ongoing surveillance in endemic regions helps identify potential new clades or variants that might pose a risk for future outbreaks^[117]^

The rise of Clade IIb and Ib mpox highlights the importance of genomic surveillance in tackling newly evolving outbreak situations. Through genomic analysis, researchers swiftly identified that the outbreak was driven by a unique clade with increased capabilities for human-to-human transmission[118]. Moreover, these genomic insights uncovered specific mutations that appeared to facilitate the virus’s adaptation to human hosts, especially in networks of close social and sexual contact[119]. The international sharing of genomic data played a pivotal role in fostering global cooperation, allowing nations to coordinate their responses and track the spread of the virus in real time[1].

## Future directions

First, initiatives should focus on enhancing laboratory capacity in African nations and other impacted regions. This entails expanding the variety of labs with the necessary tools for mpox PCR testing and gene sequencing. Collaborations with international organizations and increased government funding are essential for achieving this[19]. Second, to increase the viability of specimens and expedite their transfer to testing facilities, it is crucial to enhance specimen collection and transportation. This can be achieved by equipping medical facilities with important items such as refrigerators and reliable power sources, as well as by establishing effective transportation systems. Third, it is imperative to enhance infection prevention and control measures, especially in rural health facilities. This requires proper training and an adequate supply of techniques and materials to contain the spread of mpox and safeguard healthcare workers. Furthermore, access to diagnostics, vaccines, and therapeutics needs to be improved in affected regions to respond to the outbreak effectively. Addressing inequities between developed and developing nations is essential in ensuring a comprehensive global health response[120]. Routine examination for mpox should also be included in disease spectrum assessments for patients undergoing genitourinary evaluation to increase awareness and early detection[23].

Better data management systems and improved surveillance capabilities are essential for timely outbreak detection and effective decision-making to control the spread of mpox[22]. Continual genetic surveillance, analysis, and publication of new mpox cases are critical for the global community to better understand the virus and develop appropriate responses[86]. Future studies are necessary to reveal the effects of region, animal population, and human interaction on mpox. Identification of potential reservoirs, seasonal effects on disease incidence, travel status, and age distribution at mpox infection are also important[121].

Additionally, vaccination strategies play a crucial role in preventing and managing mpox. Prioritizing the inclusion of mpox virus primers in multiplex PCR assays in diagnostic laboratories can aid in rapid and accurate diagnosis. Prioritizing new vaccine delivery strategies, such as nanovaccines, can enhance the vaccine efficacy in tackling reemerging infections[122]. Vaccination of at-risk groups, including healthcare workers and immunocompromised patients, should be considered both as a post-infection and pre-infection prophylaxis[121]. Implementing these recommendations will significantly improve the management and prevention of Clade IIb mpox, mitigating its impact on public health and reducing the potential for outbreaks and global dissemination.

## Conclusion

There has been a significant spatial–temporal shift of the Mpox virus, characterized by the emergence of Clade IIb and Ib. Clade IIb has rapidly expanded globally, affecting over 100 countries, with Clade Ib also showing potential to spread globally with higher sexual transmission. This transmission requires robust surveillance and strengthened public health measures. Genetic and phylogenetic studies show that Clade IIb has limited variability and evolves more slowly than RNA viruses, influencing its transmission and adaptability. Continuous genomic monitoring is crucial to identify any genetic changes that may impact public health responses. A comprehensive approach, combining enhanced surveillance, genomic monitoring, targeted treatments, and vaccination campaigns, is crucial for controlling mpox reemergence. Understanding the distinct characteristics and evolutionary potential of different mpox clades is essential for developing effective public health strategies and preparing for future outbreaks.

## Data Availability

No new data were generated.
